# Head impact forces in rugby tackles are influenced by tackler position and the ball carrier instantaneous speed at contact in front-on, one-on-one tackle scenarios

**DOI:** 10.1016/j.jsampl.2025.100121

**Published:** 2025-11-26

**Authors:** Suzi Edwards, Andrew J. Gardner, Kenneth L. Quarrie, Timana Tahu, Gordon W. Fuller, Gary Strangman, Grant L. Iverson, Ross Tucker

**Affiliations:** a Sydney School of Health Sciences, The University of Sydney, Camperdown, NSW, Australia; b New Zealand Rugby, 100 Molesworth Street, Thorndon, Wellington, New Zealand; c Emergency Medicine Research in Sheffield Group, School of Health and Related Research, University of Sheffield, Sheffield, UK; d Neural Systems Group, Massachusetts General Hospital, Harvard Medical School, Charlestown, MA, USA; e Department of Physical Medicine and Rehabilitation, Harvard Medical School, Boston, MA, USA; f Department of Physical Medicine and Rehabilitation, Spaulding Rehabilitation Hospital and the Schoen Adams Research Institute at Spaulding Rehabilitation, Charlestown, MA, USA; g Mass General for Children Sports Concussion Program, Boston, MA, USA; h World Rugby, Pty (Ltd), Dublin, Ireland

**Keywords:** Motion capture, Head, Concussion, Biomechanics, Acceleration

## Abstract

**Background:**

It is not well understood how tackling technique influences the inertial head kinematics of a tackler or ball carrier. This study identified the modifiable components of the tackler's technique (including instantaneous speed at contact) that predicted inertial head kinematics of a male tackler and ball carrier during a front-on, one-on-one, ‘slow speed’ rugby tackle.

**Methods:**

Three-dimensional motion capture recorded 455 torso tackles across 15 rugby players. Principal component analysis identified four significant tackle related variables: *‘flexed head-kyphotic posture’*; *‘body height lowering strategy’*; ‘*instantaneous speed at contact—ball carrier*’; ‘*instantaneous speed at contact—tackler*’.

**Results:**

The ball carrier's instantaneous speed at contact, not the tackler, predicts inertial head kinematics of the tackler's and ball carrier's inertial head kinematics (p ​< ​0.05). Tacklers that adopt a more *‘flexed head-kyphotic posture’* (i.e., they were looking downwards towards the ground, adopted a kyphotic posture), resulted in higher inertial head kinematics for the tackler but lower inertial head kinematics for the ball carrier (p ​< ​0.001). A tackler with a *‘body height lowering strategy’* resulted in higher inertial head kinematics for both players (p ​< ​0.001) by tilting their pelvis forward to primarily flex their hips, and adopting an upright trunk posture with a neutral lordotic posture.

**Conclusion:**

To reduce the tackler's peak inertial head kinematics, the tackler could adopt a tackle strategy that looks upwards (i.e., not looking to the ground) and adopts partially bent-at-waist (i.e., is not upright) and avoids a kyphotic posture or tilting their pelvis forward to primarily flex their hips to lower their body height.

**Clinical trial registration:**

This is not a clinical trial.

## Graphical abstract

**Figure:**
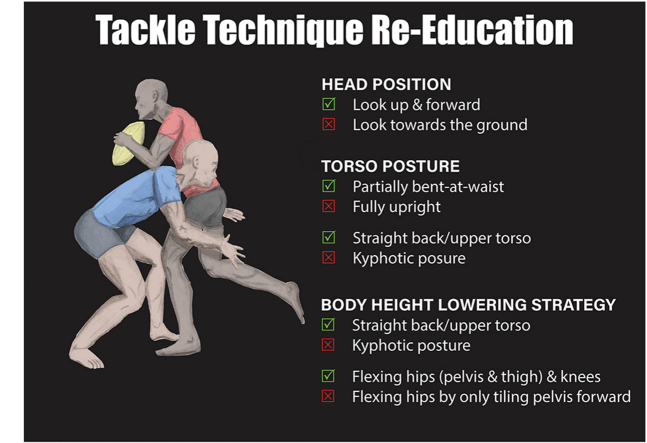

**Key points**

• A faster ball carrier instantaneous speed at contact estimates higher inertial head kinematics of both players, but a faster tackler instantaneous speed at contact only predicts higher ball carrier inertial head kinematics.
• When a tackler engages in torso contact at any height while looking towards the ground (i.e., not maintaining a posture in which their head is up and forward) or in a kyphotic posture (i.e., not maintaining a straight back/upper torso posture), it increases the magnitude of their inertial head kinematics but decreases the magnitude of the ball carrier inertial head kinematics.
• Tacklers should adopt a partially bent-at-waist posture but should not achieve this posture to lower their body height by just tilting their pelvis forward as their primary strategy to flex their hip (i.e., they should also flex their thigh segment and knee joint to lower their body height) as it predicts peak head angular acceleration for both players.
• The viability and effectiveness of tackle retraining to reduce concussion in the rugby codes requires further investigation.

## 1 Introduction

The tackle is an integral part of collision sports like rugby league and rugby union (hereafter ‘rugby’). It is the most common game play event in rugby [1,2] and possesses the highest injury risk at the elite [3] and amateur levels [4]. One of the most common tackle-related injuries is concussion [[5], [6], [7], [8], [9]], with up to 95 ​% of concussions in professional rugby league occurring during the tackle [10]. The front-on, one-on-one tackle, at a slow tackle speed is the most performed tackle and results in the greatest number of injuries [2,11], although it doesn't carry the highest risk of injury per tackle event (propensity). The tackler has a higher propensity of sustaining a head injury when performing a front-on tackle at a fast tackle speed than the ball carrier and/or tackler running a slow speed [12].

When players engage in a tackle they are exposed to impulsive forces, incurred through rapid acceleration and deceleration, that are applied either directly or indirectly to the head, via which forces are transmitted to the brain [13]. Such accelerations place strain on tether points within the brain that may lead to axonal stress, neuroinflammation, and a neurometabolic cascade [13]. How tackling technique may influence the head impulse forces (i.e., inertial head kinematics) of a tackler or ball carrier is not well understood at present. Irrespective of whether a concussion injury mechanism is a result of a single event or an accumulation of incidents [14], reducing the magnitude and frequency of impulse forces in tackles, will likely reduce the tackle-related concussion risk.

Accurately quantifying in-game 3D inertial head kinematics and the tackle technique has not been possible to date [15]. Tackle techniques and their relationships to head injury assessment (HIA)/concussion risk [[16], [17], [18]] have relied exclusively on 2D video match analysis by retrospectively reviewing known injury events [19]. The “gold standard” for evaluating accurate 3D movement [20] including inertial head kinematics is 3D optoelectronic motion capture in the laboratory. Emerging 3D movement evidence shows that during a one-on-one, front-on tackle [[21], [22], [23], [24]], the tackler's and ball carrier's inertial head kinematics can be altered by changing the instructions players receive regarding tackle techniques [23,24]. We have previously shown that when the tackler contacts the ball carrier around the mid torso region, rather than lower torso, it produces the lowest inertial head kinematics [23]. The ball carrier experiences greater inertial head kinematics when tackled at the upper torso, compared to a low [21] or mid/low torso [22] tackle height. When a tackler adheres to the tackle instruction, they increase the ball carrier's but not the tackler's inertial head kinematics [24]. If, as some research indicates, the risk of a player sustaining a concussion increases with accumulated multiple tackle events [14], then reducing the magnitude and frequency of impulse forces of the most common speed of tackles (i.e., slow speed tackles) will also assist in reducing the tackle-related concussion risk.

Given this developing evidence, the aim of this study was to identify the key modifiable components of the tackler's technique (including instantaneous speed at contact) that predicted inertial head kinematics of a male tackler and ball carrier during a front-on, one-on-one, ‘slow speed’ rugby tackle, using 3D optoelectronic motion capture. To achieve this aim we used our dataset [23] that modified key components of the tackle technique (see video Edwards et al. [25]) through changing the tackle instructions provided to players that altered the contact area on the ball carrier (upper torso, mid torso, low torso/hip area), the body region with which the tackler contacts the ball carrier (shoulder, chest/pectoral), torso position of the tackler at contact (upright, partially bent at waist, fully bent at waist), and the tackler's head position at contact.

## 2 Methods

### 2.1 Participants

Active amateur or semi-professional uninjured male rugby league (n ​= ​2), rugby union (n ​= ​8), or players of both codes (n ​= ​5) were recruited from local competitions (age 24.3 ​± ​6.1yrs, height: 1.8 ​± ​0.1 ​m, body mass: 91.4 ​± ​12.8 ​kg). The level of competition played ranged from national (n ​= ​1), state (n ​= ​2), regional (n ​= ​3), and club rugby (n ​= ​9). Participant playing experience was 12.7 ​± ​6.3 years (range 4–30 years). Both backs (n ​= ​7) and forwards (n ​= ​8) were recruited. Each participant provided written informed consent prior to their participation. The Institution's Human Research Ethics Committee (H-2017-0285) approved the study design and methodology.

### 2.2 Experimental procedures

Each data collection session involved two participants, one allocated to a ball carrier role, the other participant to the tackler role, with the participants swapping roles at the mid-point of the session. Anthropometric measurements for each participant were recorded. To modify the key components of the tackle technique that may alter inertial head kinematics, an expert coach instructed participants to perform a set of 10 trials of four different types of front-on, one-on-one torso tackles (Table 1). The expert coach was a retired dual international rugby league and rugby union player with junior and senior rugby league and rugby union coaching experience. The instructions consisted of a verbal and visual explanation of the categorisation of each of the four tackle types, including how the key modifiable tackle technique components differ between tackle types, and how, when, and why a tackler would employ each tackle type in a game (Supplementary Video [25]). Two of these tackle types followed the National Rugby League (NRL) coaching manual [26]; the tacklers' performed: (1) a ‘Smother’ tackle, where the tackler uses their chest and wraps both arms around the ball-carrier [27] to go ‘over the ball,’ and contact the ball carrier's upper torso with their head pressing into the ball carrier's shoulder [Smother NRL]); and (2) a ‘Dominant’ tackle (also known as a ‘shoulder’ tackle [27]), where the tackler makes contact with the ball carrier's hip area, in a fully bent over trunk posture *[Dominant NRL]*. The two other tackling techniques employed in this research program were variants of the traditional NRL tackles. The key differences between the traditional technique and the variant were: (3) in the ‘Smother’ tackle (i.e., *Smother Ball Pop and Lock*) the tackler lowered their contact to the ball carrier's mid-upper torso (base of chest) with their head outside the ball carrier's shoulder, and then perform a vertical ‘pop up’ action to reorient the motion of the ball carrier into an upward direction; and (4) in the ‘Dominant’ tackle (i.e., *Dominant, Torso and Stick*) the tackler makes contact with the ball carrier's mid-torso area in a partially bent over trunk posture.

**Table 1:** Categorisation of the tackling instruction.

Tackle Information	*Smother Tackle (i.e., over the ball)*		*Dominant Tackle (i.e., under the ball)*	
Tackle Type	*Smother NRL*	*Smother Ball, Pop & Lock*	*Dominant, Torso & Stick*	*Dominant NRL*
Tackle objectives.	Prevent the ball carrier offloading the ball and/or wrap up the ball and ball carrier together to (i) prevent the ball carrier using his forearm/ball as a defensive strategy to bump off the tackler and (ii) control the ball carrier when taking the tackle to the ground to slow the play the ball.		By placing ball carriers on their back, it enables the defensive team a longer duration to get back onside for next play. This tackle also aims to move the ball carrier laterally instead of driving the ball carrier backwards to allow defensive teammates to assist in the tackle.	By placing ball carriers on their back, it enables the defensive team longer duration to get back onside for the next play. This tackle also aims for the tackler to dip under the ball carrying side of the attacker and drive the attacker backwards to be dominant in the tackle.
Contact area on the ball carrier as defined by Tierney et al. [45].	Upper torso (base of chest/pectorals to line of the shoulders).	Mid-Upper torso (base of chest).	Mid torso (top of pelvis and base of chest/pectorals).	Hip area (base of pelvis to the top of pelvis).
Tackler makes contact with:	Shoulder.	Chest/Pectoral region.	Shoulder.	Shoulder.
Tackler torso position (in the sagittal plane) as defined by Stokes et al. [8].	Upright/partially bent at waist∗ (∗dependent on the vertical height of ball carrier and tackler, and where the ball carrier is holding the ball).	Partially bent at waist.	Partially bent at waist.	Fully bent at waist.
Tackle engagement description.	Tacklers use their chest and wrap both arms around the ball carrier as defined by Fuller et al. [2].	Tacklers use their pectoral on the ball carrier's forearm and ball to wrap both their arms around the ball carrier (i.e., tackler right pectoral, ball carrier's right forearm/ball).	Tacklers use their shoulder to engage in contact with the ball carrier's abdomen (i.e., mid torso; tackler right shoulder, ball carrier's non-ball carrying side).	Tacklers use their shoulder to engage in contact with the ball carrier's lower torso.
Tackle execution description.	Tacklers position themselves underneath the ball, and then execute the tackle.	Tacklers lower the vertical height of their body position and then perform a vertical *‘pop up*’ action to reorient the motion of the ball carrier into an upward direction.	Tackler moves the ball carrier in a *backward and upward* direction during the tackle and places the ball carrier on his back at the completion of the tackle.	Tackler moves the ball carrier in *backward* direction during the tackle and places the ball carrier on his back at the completion of the tackle as defined by King et al. [46].
Tackler head position.	Tacklers position their heads *within* the ball carrier's shoulder when engaging in contact and may not avoid the ball carrier using his forearm/ball to bump the tackler away.	Tacklers position their heads *outside* the ball carrier's shoulder when engaging in contact.	As the tackle is executed on the non-ball carrying side, the tackler's head is away from the ball carrier's forearm, which avoids the ball carrier using his forearm/ball to bump the tackler away.	Tacklers duck their heads prior to contact and may end up beside or in front of the ball carrier depending on the movement of the ball carrier prior to the contact.
Gaze direction as per Hendricks et al. [47].	Up and forward, gaze focused on ball carrier.	Up and forward, gaze focused on ball carrier.	Up and forward, gaze focused on ball carrier.	Down, gaze pointing towards the ground (not the ball carrier).
When to employ tackle (ball carrying side).	Not specified.	Ball carrier is holding ball on same side as tackler (i.e., ball in right hand of ball carrier, tackler is to the left side with respect to the ball carrier, and performs the tackle with his right side).	Ball carrier is holding ball on the opposite side as tackler (i.e., ball in left hand of ball carrier, tackler is to the left side with respect to the ball carrier and performs the tackle with his right side).	Not specified.
Traffic light system defined by Tackle Safe Program [48].	Red zone.	Amber/Red zone.	Amber zone.	Green zone.
Revised Traffic Light System as per this study's finding.	Orange zone – Tackler.	Green zone – Tackler.	Green – Tackler.	Red zone – Tackler.
	Orange zone – Ball carrier.	Red zone – Ball carrier.	Orange zone – Ball carrier.	Green zone – Ball carrier.

All front-on, one-on-one torso tackle trials were performed on a matted surface. Trials started with both participants stationary, 4 ​m apart. They were instructed by an expert coach to run towards each other and engage in contact at 80 ​% game intensity [28]. This led to a tackle in which, immediately pre-contact, the instantaneous speed of the tackler (2.46 ​± ​0.54 ​m/s) and ball carrier (3.15 ​± ​0.40 ​m/s) was similar to, although slightly slower than, the instantaneous speed pre-contact of the tackler (2.82 ​± ​1.07 ​m/s) and ball carrier (4.73 ​± ​1.12 ​m/s) reported in a rugby league game [29].

Previous research has indicated that player-on-player contact carries greater risks of injury in tackle situations than player-to-ground contact [11], we focussed on the player impact, and did not require the tackle to be completed to ground.’ Each set of 10 tackles comprised five dominant shoulder engagements by the tackler followed by five trials with non-dominant shoulder side engagements by the tackler. After the 40 tackle trials were completed, the roles of the participants were swapped. Rest periods comprised of 30-s between trials and ∼5-min rest between sets of each tackle type. Total session duration was ∼3-h.

### 2.3 Data collection and analysis

The 3D motion data collection and analysis procedure is described in-depth in the Supplementary Material in Edwards, Tucker, Quarrie, Tahu, Gardner [25]. Recording the full-body 3D kinematics of both participants for each tackle trial was completed using Qualisys Track Manager software (v.2018.1, Qualisys AB, Göteborg, Sweden) with a 15 Oqus 700+ camera optoelectronic motion capture system (300 ​Hz; data collection volume 10 ​m−10 ​m−6 ​m). This Qualisys 3D optoelectronic motion capture system used within this study has a reported accuracy of 0.200 ​mm (SD 0.098 ​mm) [30], which is a similar accuracy of <1 ​mm in helmeted acceleration testing accuracy using a Vicon 3D optoelectronic motion capture system [31]. 3D data analysis was performed using Visual 3D software (v6, C-Motion, Germantown, USA). Data processing began with the raw kinematic data being interpolated with a cubic spline and then filtered by a zero phase fourth order low pass Butterworth digital filter prior to calculating kinematic variables (*f*_*c*_ ​= ​18 ​Hz).

To calculate the inertial head kinematics of both players, the head segment was modelled as an ellipsoid shape [25] and the head centre of gravity linear (g) and angular (°/s) acceleration were calculated via double differentiation of the 300 ​Hz positional data. Like any technology the limitations of the measurement devices should be borne in mind when the data collected using them are considered (Supplementary 1).

### 2.4 Statistical analysis

We deemed a sample size of 14 participants to be sufficient based on the tackler's trunk flexion at contact when performing an upper, and lower, torso tackle [32]. The power calculation was determined for two-tailed t-test with an error probability and statistical power of 95 ​% using G∗Power software. Of the 600 trials collected across 15 participants, 145 trials were considered not to adhere to the tackle instruction guidelines (Table 1) and discarded from the analysis; this data is explored in Edwards et al. [24]. A trial was excluded if the tackler did not adhere to the tackling instruction for each specific instruction and this included the tackler's head not being on the side of the ball carriers' body (right shoulder side tackle, left side of ball carriers' body). Visual 3D software (Version 6, C-Motion, Germantown, MD, USA) animation of each trial was reviewed together by two authors (the expert coach; biomechanist) and discussed in accordance to tackle technique instructions (Table 1) until consensus was achieved [24].

The trials analysed for the peak resultant head acceleration variables ranged from 428 to 439 successful cases. The tackler's rear lower limb was defined as the foot that contacted the ground at Step 2, and the lead lower limb was defined as the other foot (i.e., the foot that contacted the ground at Step 1).

All statistical procedures were performed using IBM SPSS (Version 26.0, IBM SPSS Statistics for Windows, Armonk, NY: IBM Corp). All discrete variables were checked to ensure the assumptions of sphericity and normality of distribution were satisfied. Based on our previous tackle research [28], 12 kinematic variables at contact were identified. These included: joint angles (lead and rear hip flexion/extension, lumbopelvic flexion/extension, thoracolumbar flexion/extension, trunk-pelvis flexion/extension) and segment angles (pelvis [lower torso]], lumbar [mid torso], thoracic [upper torso], thorax [mid and upper torso], head flexion/extension), and the resultant instantaneous velocity of the centre of gravity of the ball carrier and tackler. An evaluation of the Pearson-product moment correlation matrix identified that these 12 variables and the inertial head kinematics of the tackler and ball carrier were significantly correlated (Table 2). As the assumption of non-collinearity was violated, a principal component analysis was employed to transform the 12 variables into uncorrelated principal components [33], using a varimax rotation. Four principal components with eigenvalues above 1.0 were identified, and the variables in each principal components were identified using a criterion of 0.50. These four principal components were then categorised as tackle technique categories based on the loadings of variables on each principal component, as follows: *1) “flexed head-kyphotic posture”, 2)* “*body height lowering strategy*”*,* and the *speed at contact* of the 3) ball carrier and 4) tackler (Table 2).

**Table 2:** Pearson-product moment correlations between the tackler's and ball carrier's peak head angular and linear acceleration with the 12 kinematic variables (range n ​= ​410 to 428) of the tackler at contact in a front-on, one-on-one tackle.

Principal Component		1	1	1	1	1	2	2	2	2	2	3	4
Variable		Thoracic	Thorax	Thoraco-lumbar	Lumbar	Head	Lumbo-pelvic	Lead hip	Rear hip	Thorax-pelvis	Pelvis	Tackler resultant speed at contact	Ball carrier resultant speed at contact
Peak head angular acceleration (Tackler)	r	−0.067	-.124∗	.163∗∗	.148∗∗	-.110∗	-.348∗∗	.315∗∗	.296∗∗	.177∗∗	-.210∗∗	0.055	.259∗∗
Peak head linear acceleration (Tackler)	r	-.271∗∗	-.310∗∗	.327∗∗	0.057	-.290∗∗	-.280∗∗	.358∗∗	.303∗∗	0.014	-.280∗∗	0.050	.232∗∗
Peak head angular acceleration (Ball Carrier)	r	.258∗∗	.319∗∗	0.012	.402∗∗	.136∗∗	-.486∗∗	.158∗∗	.202∗∗	.501∗∗	−0.084	.206∗∗	.199∗∗
Peak head linear acceleration (Ball carrier)	r	.268∗∗	.331∗∗	−0.021	.353∗∗	.202∗∗	-.371∗∗	0.042	0.047	.417∗∗	−0.032	.274∗∗	.200∗∗

A generalised linear mixed model was then used to determine 3D tackle technique categories that estimated inertial head kinematics [34]. A multiple regression was not employed in this study as it would not account for the within-subject effects in the clustered data, the process leads to errors in the estimation of parameter estimates and inflates the goodness of fit [34]. In the mixed linear model, the four tackle technique categories were input as the fixed effects and the participant number (n ​= ​15), shoulder side dominance (n ​= ​2), tackle instruction (n ​= ​4), and count (n ​= ​5; i.e., the number of trials per condition), were input as the random effects. Non-significant predictors (p ​> ​0.05) were sequentially removed by backwards elimination, such that the predictors in the full model that contributed the least were removed and this process was repeated until all predictors in the model were statistically significant.

## 3 Results

Means ​± ​standard deviations of the tackler and ball carrier inertial head kinematics, instantaneous speed at pre-contact and contact, and the 12 kinematic variables of the tackler at contact are listed in Table 3.

**Table 3:** Means ​± ​standard deviations (SD) of the tacklers and ball carrier peak head angular and linear acceleration, and the 12 kinematic variables of the tackler at contact in a front-on, one-on-one tackle.

Variable	N	Mean	±	SD
Tackler peak head angular acceleration (rad/s)	428	366.08	±	174.24
Tackler peak head linear acceleration (G)	428	5.01	±	2.14
Ball carrier peak head angular acceleration (rad/s)	439	231.90	±	125.52
Ball carrier peak head linear acceleration (G)	438	3.96	±	1.73
Tackler resultant instantaneous speed at contact (m/s)	451	1.92	±	0.49
Tackler resultant instantaneous speed at pre-contact (m/s)	451	2.46	±	0.54
Ball carrier resultant instantaneous speed at contact (m/s)	451	2.36	±	0.54
Ball carrier resultant instantaneous speed at pre-contact (m/s)	452	3.15	±	0.40
Tacklers body position at contact				
Head extension (+)/flexion (−) segment angle (°)	453	−37.80	±	22.28
Trunk-pelvis extension (+)/flexion (−) joint angle (°)	454	−41.77	±	19.15
Thorax extension (+)/flexion (−) segment angle (°)	454	−63.81	±	16.83
Thoracolumbar flexion (+)/extension (−) joint angle (°)	440	16.89	±	16.28
Lumbopelvic flexion (+)/extension (−) joint angle (°)	454	21.75	±	19.93
Thoracic extension (+)/flexion (−) segment angle (°)	438	−61.51	±	21.80
Lumbar extension (+)/flexion (−) segment angle (°)	454	−43.72	±	13.36
Pelvis posterior (+)/anterior (−) inclination segment angle (°)	454	−18.13	±	20.27
Lead hip flexion (+)/extension (−) joint angle (°)	449	77.59	±	19.98
Rear hip flexion (+)/extension (−) joint angle (°)	450	64.15	±	26.15

**Fig. 1:**
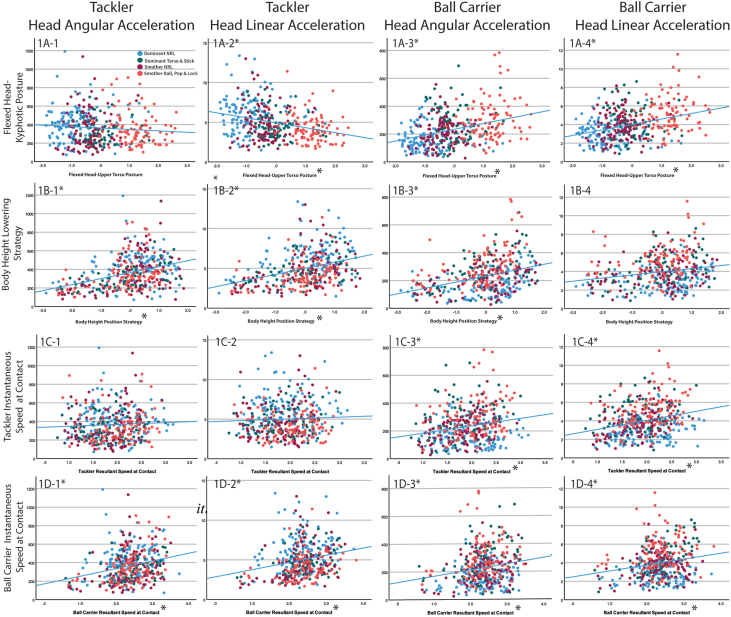
Scatterplot of the inertial head kinematics relationship of the tackler and the ball carrier to the four tackle technique categories.

**Fig. 2:**
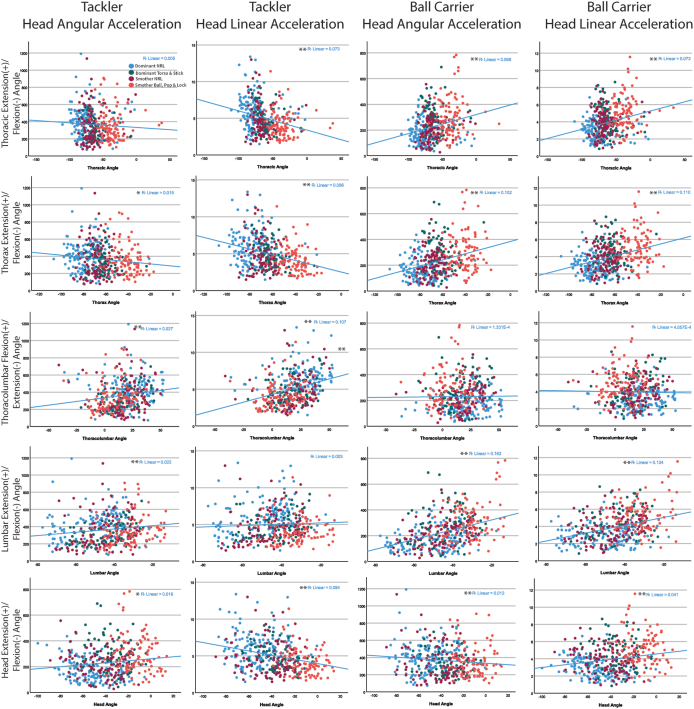
Scatterplots of the tackler's and ball carrier's peak head angular and linear acceleration with the five kinematic variables of the tackler that comprise of *“flexed head-kyphotic posture”* at contact in a front-on, one-on-one tackle.

Four tackle technique categories were identified in the principal component analysis (Table 4). The “*flexed head-kyphotic posture”* category comprised of five variables of the head and upper body posture (head, thoracic, lumbar and thorax segment angles, and the thoracolumbar joint angle [thoracic segment relative to lumbar segment]). When this principal component variable value is negative (on the x-axis, Fig. 1−A1−A4]), it reflected a more flexed head/upper torso posture. The relationships between these five variables that were computed into the *“flexed head-kyphotic posture”* variable and the inertial head kinematics are shown in Fig. 2.

**Table 4:** Principal component analysis of the 12 kinematic variables of the tackler at contact in a front-on, one-on-one tackler.

Variable	Principal Component (i.e., tackle technique categories)			
	1	2	3	4
Thoracic segment angle	**0.953**	−0.034	−0.030	−0.055
Thorax segment angle	**0.951**	−0.101	0.159	−0.018
Thoracolumbar joint angle	**−0.666**	0.226	0.494	0.350
Lumbar segment angle	**0.625**	0.231	0.559	0.263
Head segment angle	**0.578**	−0.296	0.493	0.022
Lumbopelvic joint angle	−0.271	**−0.878**	−0.218	−0.258
Rear hip joint angle	−0.227	**0.797**	0.063	−0.199
Lead pip joint angle	−0.364	**0.768**	0.006	0.302
Trunk-pelvis joint angle	0.674	**0.674**	−0.010	0.074
Pelvis segment angle	0.024	**−0.603**	0.327	0.308
Ball carrier resultant instantaneous speed at contact	0.012	0.024	**0.831**	−0.184
Tackler resultant instantaneous speed at contact	−0.042	0.000	−0.111	**0.916**

The principal component units for angles are degrees squared, and for resultant instantaneous speed are meters per second squared.

**Fig. 3:**
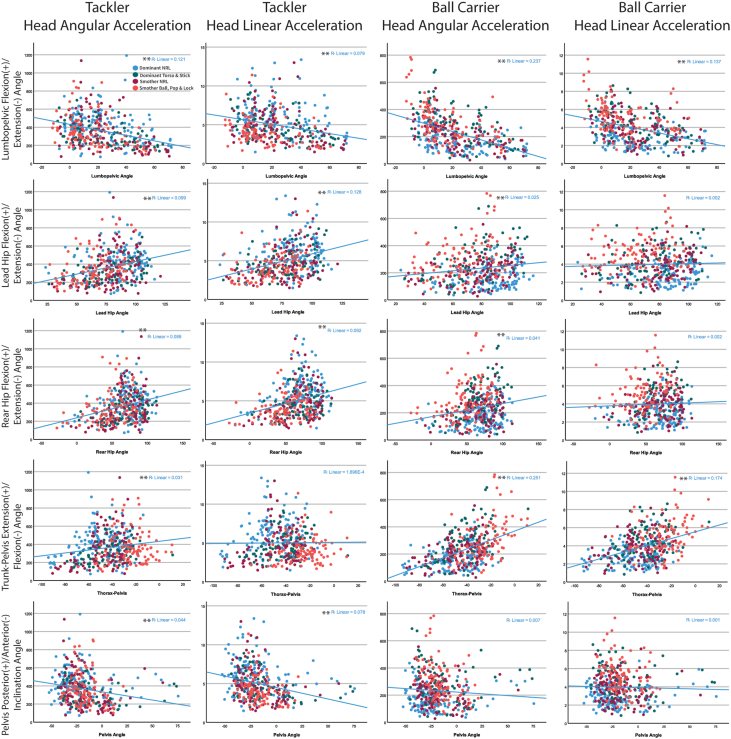
Scatterplots of the tacklers and ball carrier peak head angular and linear acceleration with the five kinematic variables of the tackler that comprise of *“body height lowering strategy’”* at contact in a front-on, one-on-one tackle.

The tackler's *‘body height lowering strategy’* category comprises of the angles of the tackler at contact of the pelvis segment, lead and rear hip (thigh segment relative to pelvis segment), lumbopelvic (lumbar segment relative to pelvis segment), and trunk-pelvis (thorax segment relative to pelvis segment) joint angles. The pelvis segment is the common segment in the calculation of the four angles that form the tackler's *‘body height lowering strategy’* and the interconnecting segment between the upper and lower extremities. When this principal component variable value is positive (on the x-axis, see Fig. 1−B1–B4), the lower trunk and hip angles were in a more flexed posture. The relationships between these five variables that were computed into the *“body height lowering strategy”* variable and the inertial head kinematics are shown in Fig. 3.

The speed of the ball carrier and tackler were the other two tackle technique categories predicted in the principal component. The relationships between the inertial head kinematics and each of the four tackle categories are presented in scatterplots in Fig. 3.

A higher peak head angular acceleration (400/455 successful trials) for the tackler was predicted with a lower *“body height lowering strategy”* (p ​< ​0.001, 95%CI 31.1−87.0, Fig. 1B-1), and a faster instantaneous speed of the ball carrier at contact (p ​= ​0.002, 95%CI 21.5−95.6, Fig. 1D-1). An increase in the tackler's peak head linear acceleration (400/455 successful trials) was predicted by a more “*flexed head-kyphotic posture”* (p ​< ​0.001, 95%CI 0.20−0.97, Fig. 1, Fig. 2), and lower *“body height lowering strategy”* (p ​= ​0.003, 95%CI -0.72−-0.25, Fig. 1, Fig. 2), and a faster instantaneous speed of the ball carrier at contact (p ​= ​0.043, 95%CI 0.01−0.90, Fig. 1, Fig. 2).

A higher peak head angular acceleration (409/455 successful trials) for the ball carrier was predicted by the tackler adopting a less “*flexed head-kyphotic posture”* (p ​< ​0.001, 95%CI 14.0−43.0, Fig. 1, Fig. 2, Fig. 3), lower *“body height lowering strategy”* (p ​= ​0.008, 95%CI 7.3−49.0, Fig. 1, Fig. 2, Fig. 3), and faster instantaneous speed of both the ball carrier (p ​< ​0.000, 95%CI 42.1−86.8, Fig. 1, Fig. 2, Fig. 3), and the tackler at contact (p ​= ​0.047, 95%CI 0.3 to 52.8, Fig. 1, Fig. 2, Fig. 3). Peak head linear acceleration (410/455 successful trials) was predicted by a less “*flexed head-kyphotic posture”* of the tackler (p ​< ​0.001, 95%CI 0.38−0.77, Fig. 1A-4), and a faster instantaneous speed of the ball carrier (p ​< ​0.001, 95%CI 0.49−1.12, Fig. 1D-4) and tackler at contact (p ​= ​0.005, 95%CI 0.15−0.93, Fig. 2C-4).

## 4 Discussion

This study utilised a large 3D mocap dataset of successful legal, front-on, one-on-one torso rugby tackles to continue to advance the mechanistic understanding of how tackle technique influences inertial head kinematics. This study included a number of tackle technique components not previously reported in research studies including the contact area on the ball carrier (upper torso, mid torso, hip area), the ball carrier's body part where the tackler made the initial contact (shoulder, chest/pectoral region), the torso position of the tackler at contact (upright, partially bent at waist, fully bent at waist), and the tackler's head position at contact. These were the 12 ​at-contact kinematic variables that may alter inertial head kinematics, based on our previous work [28]. A principal component analysis was used to transform the significantly correlated at-contact kinematic variables into four tackle technique categories and assess their ability to predict inertial head kinematics.

Similar instantaneous speeds at contact (median 1.94 ​m/s vs 2.37 ​m/s) were seen for the tackler and ball carrier. The tackler has been reported to be at a greater risk of an injury [11], including greater risk of concussion [9] or suspected concussion (i.e., a player removed from play for a HIA) [12,35] when the tackler enters the tackle at high speed. At the slow tackle speeds enforced in the study protocol the tackler's instantaneous speed at contact did not predict their peak linear or angular head acceleration. This contrary to this previous research measuring average speed via qualitative categorisation of speed [12] or computational modelling simulation [36] that showed that the tackler's magnitude of inertial head kinematics increased as their speed increased. This study did identify that the ball carrier engaging in contact at a faster instantaneous speed predict higher peak head linear and angular acceleration of both the tackler and the ball carrier. This study supports previous research in computational modelling simulation in front-one, one-on-one tackle [36] but does not support previous research that has shown that the concussion risk for the tackler when entering into a tackle at a high average speed is contingent upon the ball carrier speed into contact [12]. That is, when the tackler average speed is high and the ball carrier is static or a slow average speed, the concussion risk is increased, and the concussion risk decreases when the average speed of the tackler and ball carrier is both high [12]. This study highlights that when both the tackler and ball carrier engage in contact with an instantaneous speed categorised as a ‘slow tackle speed’ at contact, the instantaneous speed at contact predicts inertial head kinematics, however, it is the ball carrier speed at contact that has the most influence on the magnitude of the tackler's inertial head kinematics.

When a tackler adopted a more *“flexed head-kyphotic posture”* it was observed that they were not maintaining the head up and forward position (i.e., they were looking downwards towards ground and not seeing what they are going to hit), nor a straight back upper torso posture (i.e., they adopted a kyphotic posture); this resulted in a significantly higher peak head linear acceleration for the tackler. This finding highlights the importance for coaches to ensure the tackler maintains a head up and forward position [16,17] and straight back posture [16] as these two variable both independently increase the tackler's risk for a HIA in a front-on tackle and are associated with tackle performance [37]. Adopting a *“flexed head-kyphotic posture”* may also adversely affected tackle performance, by ineffectively imparting an external force onto the ball carrier's body, as evidenced by this posture having a significantly lower ball carrier peak head angular acceleration.

Higher linear and angular head acceleration for the tackler and angular head acceleration for the ball carrier was also predicted by the tackler's *‘body height lowering strategy’*. Higher inertial kinematics were estimated when the tackler's *‘body height lowering strategy’* involved the tackler positioning their pelvis segment with a more anterior inclination, greater hip joint flexion, and a more neutral lumbopelvic and trunk-pelvis joint posture at contact. To simplify this, higher inertial head kinematics occurred when the tackler attained greater hip flexion, primary via an increase anterior inclination of the pelvis. The role of a hip flexion strategy and the importance of a tackler's leg drive at contact (a factor that has been shown to be associated with a decreased risk for sustaining a concussion [17,18]) warrants further investigation. Higher inertial head kinematics were predicted when the tackler combined a more anterior pelvis inclination with a more neutral lumbopelvic and trunk-pelvis posture to engage in torso contact with the ball carrier; a strategy that suggests that the tacklers are adopting a more upright body position in a more neutral lordotic posture. Adopting an upright body position compared to a bent at waist position is associated with a higher propensity for a HIA in professional rugby union [12] and rugby league [38], tackle performance in rugby union [37] and is advocated in injury prevention programs such as the Boksmart program [39]; the current study highlights that it is critical to consider the way the pelvis and trunk segments interact (i.e., how the tackler gets in to an upright or non-upright torso position) to attain spinal alignment, and the influence that it has on the inertial head kinematics magnitude. As such, it is recommended that future research avoid merging the pelvis, lumbar, and thoracic segments into a single torso segment, like many other complex 3D sporting movements studies have done [[40], [41], [42]] including our tackle research [23,24,28] compared the common single torso segment approach [32,43,44]. This approach appears likely to result in information that substantially influences the magnitude of inertial head kinematics of the tackler, being overlooked/missed.

The current study has several limitations reported in detail elsewhere [23]. In summary, the results may not generalise to modifying extrinsic risk factors of the tackle by involving more than one tackler, different directions of approach between the tackler and ball carrier, different speeds of the participants leading into, and at, contact, non-legal tackles, or tackles taken to ground or performed in game. This study's results are not generalised to other athlete groups with different intrinsic risk factors such as different player experience, differing player skill level, age, female players, or differing injury histories.

## 5 Conclusion

A faster ball carrier speed predicted with higher inertial head kinematics of the tackler and the ball carrier, whereas only the tackler's speed influenced the ball carrier's inertial head kinematics. When a tackler looks downward at the ground adopting a kyphotic posture in a front-on, one-on-one tackle, they sustained a higher magnitude of inertial head kinematics, however, this leads to the lowest inertial head kinematics for the ball carrier. This study identified the importance of the interaction between the pelvis, lumbar, and thoracic segments, which predicted a higher peak head angular acceleration of the ball carrier and tackler. Our results indicate that the lumbar [mid torso] and thoracic [upper torso] segments should not be treated as a single torso segment (i.e., thorax [mid and upper torso]), as has been the common approach of most of 3D kinematic analysis research to date.

## Patient consent statement

Each participant provided written informed consent prior to their participation.

## Data availability statement

Researchers are welcome to contact Dr Suzi Edwards the corresponding author for data sharing purposes to support the enhancement of high-quality tackle biomechanics research.

## Ethics approval statement

The study has been approved by The University of Newcastle Human Research Ethics Committee (H-2017-0285).

## Permission to reproduce material from other sources

Not applicable.

## Funding statement

We acknowledge research support from the 10.13039/501100023790National Rugby League Rugby League Research Committee (RLRC) for the examination of rugby-style tackle techniques.

## Declaration of competing interest

Suzi Edwards leads a tackle re-education program that is partially funded by a NHMRC Ideas 2021 grant (202718). Andrew Gardner, Ph.D. has a clinical practice in neuropsychology involving individuals who have sustained sport-related concussion (including current and former athletes). He has been a contracted concussion consultant to Rugby Australia since July 2016. He has received travel funding or been reimbursed by professional sporting bodies, and commercial organisations for discussing or presenting sport-related concussion research at meetings, scientific conferences, workshops, and symposiums. Previous grant funding includes the NSW Sporting Injuries Committee, the Brain Foundation (Australia), an Australian-American Fulbright Commission Postdoctoral Award, an NHMRC early research career fellowship, a Hunter New England Local Health District, Research, Innovation and Partnerships Health Research & Translation Centre and Clinical Research Fellowship Scheme, and the Hunter Medical Research Institute (HMRI), supported by Jennie Thomas, and the HMRI, supported by Anne Greaves. He has current salary and research funding through an NHMRC Investigator grant. He has received unrestricted philanthropic support from the National Rugby League (NRL) and the Nick Tooth Foundation. Timana Tahu is employed as a researcher by The University of Sydney (NHMRC Ideas 2021 grant) and as the Senior Manager for Indigenous elite pathways for the National Rugby League. Ross Tucker is a research consultant to World Rugby (Pty) Ltd, the governing body for Rugby Union globally. Grant Iverson serves or has served as a scientific advisor for NanoDx™ (formerly BioDirection, Inc.), Sway Operations, LLC, and Highmark, Inc. He has a clinical and consulting practice in forensic neuropsychology, including expert testimony, involving individuals who have sustained mild TBIs (including athletes). He has received research funding from several test publishing companies, including ImPACT Applications, Inc., CNS Vital Signs, and Psychological Assessment Resources (PAR, Inc.). He has received research funding as a principal investigator from the National Football League, and salary support as a collaborator from the Harvard Integrated Program to Protect and Improve the Health of National Football League Players Association Members. Gary Strangman has received pilot research funding from the Harvard Football Player's Health Study, funded through the National Football League Players Association. Gary Strangman has received pilot research funding from the Harvard Football Player's Health Study, funded through the National Football League Players Association. Kenneth Quarrie is employed as the Chief Scientist for New Zealand Rugby. He has received support for travel and accommodation from World Rugby for the purpose of attending World Rugby Medical Conferences and Scientific Committee meetings. He is the Co-Principal Investigator of the Kumanu Tāngata project, a study examining long-term health outcomes associated with high-level rugby participation, which is funded by World Rugby and the New Zealand Rugby Foundation.
