# Supplementary material for: Head impact forces in rugby tackles are influenced by tackler position and the ball carrier instantaneous speed at contact in front-on, one-on-one tackle scenarios
Source: JSAMS Plus. 2025 Nov 26;6:100121. doi: 10.1016/j.jsampl.2025.100121 (PMC13008422; doi:10.1016/j.jsampl.2025.100121)
Supplement: Multimedia component 1 [file mmc1.docx]

**Supplementary – Inertial head kinematics methodology**

Like any technology used for collecting data, we should be aware of the limitations of the measurement devices we have employed when capturing information. 3D optoelectronic motion capture systems are recognized as the gold standard of 3D motion capture (e.g., Vicon [Oxford, UK], Qualisys [Gothenburg, Sweden)]), and routinely used to validate the reliability of wearable sensors^1,2^, including helmet impact testing^3^ and model-based image matching^4^. This study used the Qualisys 3D optoelectronic motion capture system that has a reported accuracy of 0.200 mm (SD 0.098)^5^ and similar accuracy of Vicon 3D optoelectronic motion capture system reported accuracy of <1 mm in helmeted acceleration testing^3^. The sampling frequency of time-series data must be high enough to record the dynamics of the fastest changes in the system (i.e., to ensure you capture the entire signal) but not so high that it introduces too much high frequency noise^6^. This is why in this study we undertook *spectral analysis* of the tackler’s and the ball carrier’s raw 3D head linear centre of gravity position acceleration to identify the median frequency that captured 99% of the data (median frequency of 12 Hz; range 9 to 14 Hz). This enabled us to identify the most appropriate sampling rate for this study to ensure we were not adding additional spurious high frequency noise by oversampling the data.

**Supplementary 2– Tackle Speed**

Applying Fitts law of speed-accuracy ^7^ to the tackle event, there is a trade-off between a player’s speed and the accuracy of the tackler contacting the entry point (e.g., mid torso) when engaging in contact in a tackle. Participants reduced their instantaneous pre-contact speed, then further rapidly reduced their speed by decelerating one to two steps before entering into contact (Table 1; Supplementary 6^8^). For example, the speed-accuracy trade off in this study (i.e., using derivative of positional data measured at 300 Hz) shows that a participant starting in a stationary position accelerated to reach an instantaneous speed at pre-contact of 3.36 m/s for the ball carrier and 2.62 m/s for the tackler, then both players decelerated two steps prior to contact to display slower instantaneous speeds at contact of 1.87 m/s for the ball carrier and 1.33 m/s for the tackler. Instantaneous speed was measured in this study, and not ‘average’ speed which is typically measured by either qualitative categorisation of speed^9^  or quantitative over a 0.5 second duration by 10 Hz 2D video^10^ or 10 Hz global positioning units^11^. The tackler’s (2.46±0.54 m/s) and ball carrier’s (3.15±0.40 m/s) resultant instantaneous speed at pre-contact was similar to the average speed of the tackler (2.82±1.07 m/s) and ball carrier (4.73±1.12 m/s) reported elsewhere in rugby league^11^.

**References**

1. Mayagoitia RE, Nene AV, Veltink PH. Accelerometer and rate gyroscope measurement of kinematics: an inexpensive alternative to optical motion analysis systems. *J Biomech.* 2002; 35(4):537-542.

2. Edwards S, White S, Humphreys S, Robergs R, O’Dwyer N. Caution using data from triaxial accelerometers housed in player tracking units during running. *J Sports Sci.* 2019; 37(7):810-818.

3. Joodaki H, Bailey A, Lessley D, Funk J, Sherwood C, Crandall J. Relative motion between the helmet and the head in football impact test. *J Biomech Eng.* 2019; 141(8).

4. Tierney GJ, Joodaki H, Krosshaug T, Forman JL, Crandall JR, Simms CK. Assessment of model-based image-matching for future reconstruction of unhelmeted sport head impact kinematics. *Sports Biomech.* 2018; 17(1):33-47.

5. Topley M, Richards JG. A comparison of currently available optoelectronic motion capture systems. *J Biomech.* 2020; 106:109820.

6. Skiadopoulos A, Stergiou N. Chapter 5 - Power spectrum and filtering, in *Biomechanics and Gait Analysis*. Stergiou N, ed^eds, Academic Press, 2020.

7. Schmidt RA, Lee TD. Chapter Performance and motor control characteristics of functional skills in *Motor Learning and Control: Concepts and Applications* 11th, ed^eds. New York, NY McGraw-Hill Education 2021.

8. Edwards S, Tucker R, Quarrie K, Tahu T, Gardner A. Tacklers shoulder abduction and flexion at contact alters when engaging in different front-on, one-on-one tackle instructions from an expert coach. *J Sci Med Sport.* 2024; 27(7):472-479.

9. Tucker R, Raftery M, Kemp S, et al. Risk factors for head injury events in professional rugby union: a video analysis of 464 head injury events to inform proposed injury prevention strategies. *Br J Sport Med.* 2017; 51(15):1152-1157.

10. Hendricks S, Karpul D, Nicolls F, Lambert M. Velocity and acceleration before contact in the tackle during rugby union matches. *J Sports Sci.* 2012; 30(12):1215-1224.

11. Parmley J, Jones B, Whitehead S, et al. The speed and acceleration of the ball carrier and tackler into contact during front-on tackles in rugby league. *J Sports Sci.* 2023; 41(15):1450-1458.
